# Empowering patients through clinical notes access: Opportunities, challenges, and the path forward

**DOI:** 10.1371/journal.pdig.0000993

**Published:** 2025-10-08

**Authors:** Yoko Yoshimura, Geva Greenfield, Benedict Hayhoe, Azeem Majeed, Ana Luisa Neves

**Affiliations:** Department of Primary Care and Public Health, School of Public Health, Imperial College London, London, United Kingdom; Dana-Farber Cancer Institute, UNITED STATES OF AMERICA

## Background

Electronic Health Records (EHRs) have been in use in healthcare settings since the 1970s, with global adoption increasing in the 1990s [1]. Initially, like paper-based records, EHRs were designed primarily for healthcare professionals (HCPs) to record and share health information, rather than as interactive tools for providing information to patients. However, recently, increased recognition of patient-centeredness, patients’ rights and patient demand for access to their health data has led many healthcare systems to allow patients to view all or parts of their EHRs. This shift towards greater transparency and patient involvement is seen as a crucial step in fostering patient empowerment, improving self-care and health literacy and supporting active participation in managing their health and understanding their conditions. This may disrupt traditional healthcare workflows and introduce new ways of patient–doctor interaction.

Patient access to EHRs enhances engagement in health management. A 2020 systematic review of randomized controlled trials (RCTs) [2] found positive impacts of EHR access on patient-centeredness, with improvements in patient engagement and empowerment. Patient safety also benefited, as those with EHR access experienced fewer medication discrepancies [2]. Notably, aggregated data from several RCTs focusing on patients with type 2 diabetes showed significantly better glycaemic control among those with EHR access [2]. However, findings on efficiency were mixed, and no RCTs examined timeliness or equity, highlighting key knowledge gaps in these areas [2].

Most EHR systems limit patient access to coded data, with few allowing access to clinical notes—i.e., free-text narratives in which HCPs record patient details in their own words. Clinical notes provide more detailed, personalized information than coded data, offering potential additional benefit [3] as they reflect the unique aspects of a patient’s care. Targeted sharing of clinical notes, therefore, may further empower patients, transforming them from passive recipients of care into active participants in managing their health [3,4]. Several platforms, such as *OpenNotes* in the U.S. [3] and *Journalen* in Sweden [3,5], allow patients access to their medical notes.

Whereas patients’ access to EHRs has documented benefits [2], sharing clinical notes presents additional challenges and concerns, including issues related to privacy, language interpretation and potential miscommunication.

## Advantages of providing patients access to their clinical notes

Providing patients access to clinical notes has demonstrated benefits for patient-centered care, as patients desire this continued access to enhance their confidence in managing their health, while improving communication and trust with HCPs [4] (Table 1). This access also promotes safety by helping patients understand treatments, adhere to care plans, manage prescriptions, and identify potential errors [3,6]. There is also potential to reduce health inequalities, with marginalized populations reporting improved recall, understanding, and engagement with care plans through access to clinical notes [3]. Additional potential benefits of sharing clinical notes include timely access to information [3], reduction in unnecessary consultations and phone calls by using clinical notes as an aide-memoire [7,8], and improved health outcomes in populations requiring active self-care [6,7].

**Table 1:** Potential advantages and challenges of sharing clinical notes with patients.

Advantages	Challenges
Enhances patients’ understanding of their care and treatment rationale, fostering better communication and trust with healthcare professionals.	Patients may misunderstand technical language or sensitive information, causing anxiety.
Helps reduce unnecessary consultations and phone calls by allowing clinical notes to serve as a memory aid.	Increased patient inquiries may require more time and resources from healthcare providers.
Supports marginalized populations by enhancing recall, understanding, and engagement with care plans.	There is a potential risk of exposing sensitive information inappropriately.
Provides quick and easy access to essential information.	Healthcare professionals might adjust documentation practices, which could reduce the detail and quality of clinical notes.
Helps patients identify errors and potentially avoid adverse events.	Some clinicians worry that sharing notes might confuse patients or undermine trust.
Promotes better treatment adherence in patients managing self-care, contributing to improved health outcomes.	

In the *Journalen* study in Sweden, 96.6% of patients with clinical experience rated access to clinical notes positively, with most strongly agreeing or agreeing that it was beneficial for them [5]. Another Swedish study showed that enabling patients to review their notes extends beyond the one-time information exchange during consultations, effectively “extending the visit.” This approach has helped cancer patients better understand key information about their diagnosis and prognosis [3].

## Potential challenges of providing patients access to clinical notes

While sharing clinical notes with patients offers potential benefits, several obstacles must be addressed. Some HCPs worry that accessible notes could cause patient confusion or erode trust between patients and clinicians [3]. Furthermore, there is the potential for patients to misinterpret medical terminology or sensitive information, which may result in increased anxiety, particularly among individuals with complex medical conditions or mental health challenges [3,7]. Hence, careful implementation of record sharing is needed for vulnerable groups such as those with mental health conditions or at risk from domestic abuse, for example.

The risks of inadvertently disclosing sensitive information are significant [4], and the increase in patient inquiries stemming from shared notes may demand extra time and resources from HCPs [7]. HCPs may also adjust their documentation style, potentially compromising the depth and quality of clinical notes [3]. For instance, Blease and colleagues reported that in a survey of U.S. physicians across various specialties, 22% (*n* = 168) felt that their notes were less valuable due to the practice of open notes [6]. These challenges highlight the need for a thoughtful implementation approach, carefully considering individual patient needs to integrate shared notes effectively into healthcare practices. Training programmes and guidance for HCPs on best practices for documentation in open notes contexts could help reduce issues like excessive patient inquiries due to misinterpretation.

## The way forward: What does the future hold for open records?

**Fig 1:**
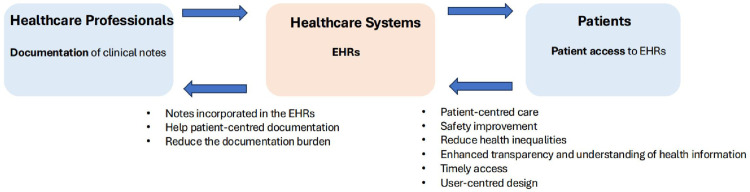
A pipeline of how clinical notes might be shared with patients.

HCPs, healthcare systems, and patients can collaborate to make the most of clinical notes sharing (Fig 1). Fig 1 demonstrates an envisioned pipeline of how clinical notes might be shared with patients—starting from documentation, through system integration, to patient access. HCPs should adopt patient-centered documentation, using clear and concise language, minimizing jargon, and offering explanations of medical terms to enhance patient understanding; this approach could help reduce the likelihood of misunderstandings and confusion. Additionally, strengthening patients’ overall health literacy may further promote effective clinical notes sharing. Ultimately, systems will need to adapt towards the development of EHR access platforms that are user-friendly, intuitive, and developed based on user-centered and co-design principles [3]. Research needs to focus on use, usability and uptake, alongside clinical effectiveness [9]. Finally, the effects of these interventions on patients may vary significantly across patient groups [9]. It is important to consider deliberate approaches to evaluate variation on adoption and impact across patients groups, including exploring interest, use and ultimate impacts on quality outcomes.

Innovations such as ambient AI scribes may assist with the summarization, translation, and explanation of clinical notes, potentially enhancing patients’ understanding of their health information. Furthermore, the integration of AI into EHRs has the potential to reduce the documentation burden on HCPs and contribute to improvements in efficiency and patient safety. However, while they may help to bridge literacy gaps, they also introduce new risks, such as the introduction and perpetuation of errors or omissions in patients’ clinical records. To understand these impacts comprehensively, longitudinal and multifaceted research is essential to assess their effects on provider workload, patient comprehension and trust and the adequacy of existing organizational safeguards.

## Conclusion

Sharing EHR clinical notes with patients potentially transforms EHRs from a clinician tool to an interactive provider–patient tool that embraces patient-centered care. While the transparency provided by clinical notes can improve patients’ care quality, addressing challenges is key for effective implementation. It is essential for HCPs and patients to actively optimize the use of clinical notes for patient benefit, collaborating through user-friendly platforms that are backed by secure systems, along with adequate training and support.
